# An Update on the Chemical Constituents and Biological Properties of Selected Species of an Underpinned Genus of Red Algae: *Chondrus*

**DOI:** 10.3390/md22010047

**Published:** 2024-01-17

**Authors:** Seon-Joo Park, Anshul Sharma, Hae-Jeung Lee

**Affiliations:** 1 Department of Food and Nutrition, College of Bionanotechnology, Gachon University, Seongnam-si 13120, Republic of Korea; chris0825@gachon.ac.kr; 2 Institute for Aging and Clinical Nutrition Research, Gachon University, Seongnam-si 13120, Republic of Korea; 3 Department of Health Sciences and Technology, Gachon Advanced Institute for Health Science and Technology (GAIHST), Gachon University, Incheon 21999, Republic of Korea

**Keywords:** *Chondrus*, Rhodophyta, seaweed, algae, anti-inflammation, antioxidant, antitumor

## Abstract

Macroalgae, particularly red seaweeds, have attracted significant attention due to their economic and health benefits. *Chondrus*, a red algae genus, despite its economic importance, seems to be undervalued. Among all its species, *Chondrus crispus* has been meticulously documented for its biological properties, and little is known about other species. No comprehensive review of the biological properties of this genus has been acknowledged. Thus, this review aimed to summarize the available information on the chemical constituents and biological properties of a few selected species, including *Chondrus crispus*, *Chondrus ocellatus*, *Mazzaella canaliculata*, and *Chondrus armatus*. We compiled and discovered that the genus is offering most of the important health-promoting benefits evidenced from in vitro and in vivo studies focused on antimicrobial, immunomodulation, neuroprotection, anti-atopic, anti-inflammatory, anti-viral, anti-diabetic, cytoprotective, antioxidant, anti-coagulation, nephroprotective, anti-tumor, and anti-venom activity, which speaks about the potential of this genus. Data on clinical studies are limited. Further, around 105 chemical constituents have been reported from *Chondrus* spp. Given its significance, further investigation is warranted, in the form of meticulously planned cell, animal, and clinical studies that concentrate on novel health-enhancing endeavors, in order to unveil the full potential of this genus. The review also outlines challenges and future directions.

## 1. Introduction

Algae are ancient photosynthetic organisms on the planet, dating back 3.5 billion years [1]. These photosynthetic species are classified as microalgae and macroalgae. Macroalgae are macroscopic, and multicellular organisms that can be categorized into three taxonomic groups, namely red algae (Rhodophyta), green algae (Chlorophyta), and brown algae (Heterokontophyta), determined by their respective color pigments. Macroalgae, also referred to as seaweeds, are macroalgal species found in marine environments, although there are also freshwater macroalgae. According to the available data, the biodiversity of red, brown, and green algae is estimated to encompass around 7000 species (most are marine), around 2100, and around 2700 species (all Ulvophyceae members are marine), respectively [2]. 

Seaweeds (macroalgae) have long been used in human diets and medicine around the world [3]. They are rich sources of proteins, lipids, carbohydrates, polysaccharides, polyphenols, essential amino acids, carotenoids, vitamins (vitamin A, B_12_, B_9_, B_2_, B_1_, C, E, D, and K), minerals (iodine (I), iron (Fe), potassium (K), zinc (Zn), copper (Cu), magnesium (Mg), and calcium (Ca)), essential fatty acids, and dietary fiber [3,4,5,6,7,8]. Important polysaccharides from marine algae include sulfated galactans, namely carrageenans, agarose, alginate, and agar from red algae, ulvans from green algae, and alginates, fucoidans, and laminarans of brown algae [9,10,11].

The chemical composition and nutritional content of the macroalgae depend upon the environmental conditions in which they thrive, thus being influenced by sunshine, temperature, pH, and carbon dioxide, which also vary among genera, species, and divisions [12]. There are reports showing that seaweeds and their extracts have been reported to be innovative sources of various bioactive secondary metabolites, including polyunsaturated fatty acids (PUFAs), carrageenan, fucoidan, etc., that have been shown to have health-promoting properties including antioxidant, anticancer, antidiabetic, antiviral, anti-obesity, anti-inflammatory, anti-aging, and antimicrobial properties [3]. Seaweed applications have been exploited in different sectors, including biomedical, cosmetics, pharmaceutical, skin, and agriculture [3]. They have garnered significant attention from the scientific community due to their huge, unexplored potential. 

According to statistics provided by the Food and Agriculture Organization (FAO), the majority of global seaweed production originates from Asia, amounting to a total of 34,826,750 tons. This figure encompasses both wild and cultivated seaweed, with the Asian region accounting for 97.38% of the total production. As per global seaweed production data for 2019, among Asian countries, China, Indonesia, and the Republic of Korea hold the first, second, and third positions in terms of seaweed production (wild and cultivated), contributing 20,296,592 tons (56.75%), 9,962,900 tons (27.86%), and 1,821,475 tons (5.09%), respectively, which corresponds to 89.7% of the world’s total share [13]. A total of five types of different varieties of seaweed species contribute to over 95% of the global seaweed culture production. These include *Laminaria* and *Saccharina*, which account for 35.33% of the production; *Kappaphycus* and *Eucheuma* (carrageen seaweeds), with a contribution of 33.46%; as well as *Gracilaria* (10.32%), *Porphyra* (8.33%), and *Undaria* (7.16%) [14]. Extensive literature is available on these five seaweed species. 

## 2. Search Strategy

A comprehensive literature review was conducted utilizing various academic databases, including Google Scholar, Embase, PubMed, and Science Direct, to identify relevant research findings from January 1995 to October 2023. A search was conducted using different keywords “Rhodophyta”, “red algae”, “*Chondrus*”, “*Chondrus crispus*”, “Irish moss”, “*Chondrus ocellatus*”, “*Chondrus canaliculatus*”, and “*Chondrus armatus*” to collect accessible literature pertaining to the genus *Chondrus*. The aim was to gather comprehensive information on the nutrients, chemical constituents, and health-promoting or biological activities of this genus. The search was also refined to include in vitro, in vivo, and clinical studies of the genus *Chondrus*. Additionally, we chose abstracts from publications published in another language, where there was not much information available about a particular species of *Chondrus*.

## 3. Rhodophyta (Red Seaweeds) 

Red seaweeds are frequently encountered in various ecosystems, encompassing marine, terrestrial, and freshwater environments, and are among the primitive groups of eukaryotic algae [15]. The class Florideophyceae encompasses a significant proportion of red algae, with the bulk of its members being multicellular organisms that mostly inhabit marine habitats [16]. The red color of these algae can be attributed to the presence of a pigment known as phycoerythrin, either alone or in conjunction with phycocyanin. These pigments, along with chlorophyll, produce the characteristic red or purple color of red algae. *Chondrus*, *Porphyra*, *Pyropia*, *Gelidium*, *Gracilaria*, *Acanthophora*, and *Palmaria* are among the common genera of red algae. Their cell walls consist of carrageenan and cellulose, the most studied compounds with potential commercial value [17]. The utilization of red algae as a valuable source of nutritional components, functional food ingredients, and therapeutic substances has been well documented throughout history [17]. They are becoming increasingly significant in the blue biotechnology industry as a source of nutrients, functional foods, feeds, and healthcare supplies. Traditionally, red algae have been consumed in its raw form and incorporated into various culinary preparations such as salads, soups, meals, and condiments. One of the genera, *Porphyra,* is eaten as a food crop in Asian countries, including Korea, Japan, and China. It is famously called gim, nori, and zicai. *Palmaria palmata*, also known as Dulse, is a highly consumed species of red algae that serves as a significant carbohydrate, protein, lipid, I, Ca, Mg, and vitamin source [18]. 

Red algae have been testified to possess health-promoting properties that encompass anti-inflammatory, anti-diabetic, antioxidant, antimicrobial, anti-hypertensive, anticoagulant, antitumor, and anti-helmintic effects. These beneficial effects can be attributed to the diverse array of compounds found in red algae, including proteins, polysaccharides, polyphenols, PUFAs, amino acids, and sterols [19]. 

Phycocolloids are polysaccharides extracted from both marine and freshwater algae. Examples of the most abundant red algae phycocolloids include carrageenans and agar. Polysaccharides, especially carrageenans, have been reported to have many beneficial activities, such as antihyperlipidemic [20], immunomodulation and anticoagulant [21], antioxidant [9], and antiviral [22]. The name “carrageenan” was coined based on the geographical origin of the raw material, namely the Irish district recognized for supplying the polysaccharide’s source material, Irish moss (*Chondrus crispus*) [23]. Carrageenans derived from red algae are classified as high-molecular-weight sulfated polysaccharides of *d*-galactose and 3,6-anhydro-*d*-galactose monomers, alternately linked by α-1,3 and β-1,4 glycosidic linkages [24]. Carrageenans make up around 40–50% of the algae’s dry weight, and they possess significant industrial potential due to their low toxicity, biodegradability, and biocompatibility [24]. There have been reports of over 10 species of carrageenans, with lambda (λ), kappa (κ), and iota (ι) being of more commercial significance [25,26]. Their classification is determined by the degree of sulfation (variations in the number and location), their average molecular mass, and the presence of a 3,6-anhydro derivative in the α galactose residues [24]. They also show differences in solubility and gelling properties [27]. Carrageenans and their derivatives have been documented to have many biological and pharmaceutical applications, including anti-oxidant, antiviral, antimicrobial, anticoagulant, anticancer, immunomodulatory, mucoadhesive, neuroprotective, tissue regeneration, and drug delivery [28,29]. Carrageenans are known for their excellent biocompatibility and can be employed independently or in conjunction with other polymers. Their use has been documented for pellets, hydrogels, films, fast-dissolving inserts, nanoparticles, beads, microparticles, intranasal systems, etc. [29]. 

Many phycocolloids, including agar, furcellaran, and carrageenan, are commonly employed as food additives, gelling agents, textiles, anticoagulants, and water-binding agents. They are produced by different species of red algae, namely *Acanthophora*, *Euchema*, *Gelidium*, *Gracilaria*, *Palmaria*, and *Porphyra* [30]. The details about Rhodophyta have been meticulously described elsewhere [16,17,31]. Precisely talking about the genus *Chondrus*, scarce information has been reported. Most of the literature is focused on one species, namely, *Chondrus crispus*. However, scattered health-promoting activities have also been reported for other species of this genus. Thus, it is essential to have compiled information on this economically important genus. Considering its importance, this review aimed to compile up-to-date information on different species names of the genus *Chondrus*, including *Chondrus crispus*, *Chondrus ocellatus*, *Chondrus armatus*, and *Mazzaella canaliculata* (C. Agardh) Arakaki & M. E. Ramírez 2021 (formerly known as *Chondrus canaliculatus*) [32], their nutrients, chemical constituents, and various health-promoting activities. After the following section, *Mazzaella canaliculata* will be used throughout the manuscript. 

## 4. General Information on the Genus *Chondrus*

The classification of the genus *Chondrus* includes the following: Kingdom: Plantae; division: Rhodophyta; subphylum (subdivision): Eurhodophytina; class: Florideophyceae; subclass: Rhodymeniophycidae; order: Gigartinales; family: Gigartinaceae; and genus: *Chondrus*. The identification of various *Chondrus* species is typically based on the morphology of their thallus and the location of their reproductive structures [33]. The peculiar cell wall of this genus is a notable attribute that contributes to its economic importance [34]. A recent study has identified genetic diversity and phylogenetic structure of three species and concluded that it was impossible to make a phenotype-based classification due to large morphological plasticity, as is characteristic of the genus [35]. It is used as a source of dietary fiber and minerals in health foods, and carrageenan is used to thicken and gel things [36]. *Chondrus* is one of several red algae genera that serves as a notable iodine source [8]. A recent study has reported 47 mg/g iodine content for *C. crispus*. Ingestion of 45 g of *C. crispus* snack was equal to 2.1 mg iodine, with a bioavailability of 50% [37]. Therefore, the *Chondrus* genus holds significance from both economic and health-enhancing perspectives. Nevertheless, the natural populations of this species have decreased due to a variety of biotic and abiotic conditions [38,39]. Many countries have started on-land cultivation that might compensate for the loss due to various factors. 

In order to evaluate the biological properties of *Chondrus* spp., researchers have evaluated the *Chondrus* spp. extracts and a few have isolated bioactive compounds to demonstrate their biological properties. The majority of investigations have employed extracts derived from various solvents and have used both dried and fresh samples of both wild and cultivated *Chondrus* spp. Extract-based extractions comprise a crude aqueous extract and an organic solvent extract, which consists of a crude ethanolic extract (95%), methanol, acidified ethanol, ethyl acetate, diethyl acetate, and varying percentages of methanolic, ethanolic, and acetone solutions. Others have utilized ultrasound-assisted extraction (UAE) and enzymatic methods for extractions. Further, a few studies have utilized different polysaccharide fractions, including carrageenan κ and λ, from *Chondrus* spp., for evaluating biological properties. Detailed information on extract types, polysaccharides, and biological applications has been provided in Table 1.

## 5. *Chondrus* Species, Chemical Compounds, and Biological Properties 

**Figure 1:**
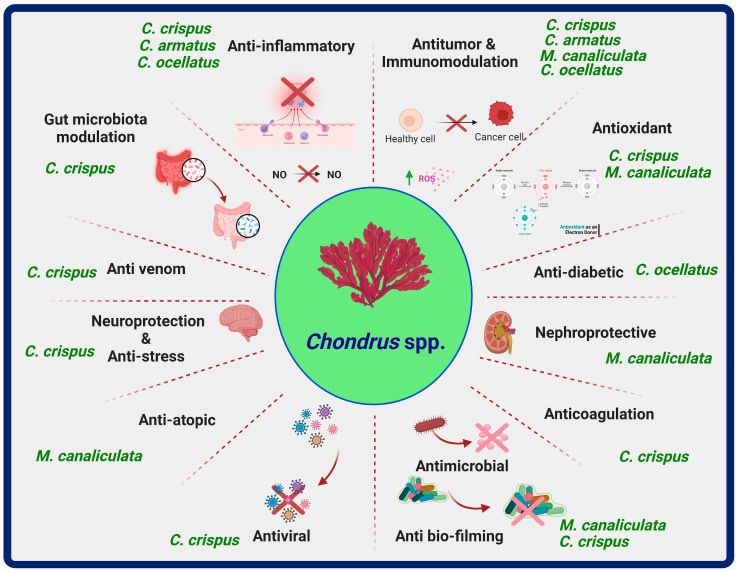
Biological properties of *Chondrus crispus*, *Chondrus ocellatus*, *M. canaliculata*, and *Chondrus armatus*. The image shows the recently accepted name (*Mazzaella canaliculata*) for *Chondrus canaliculatus*.

In the subsequent sections, detailed information on selected *Chondrus* species, specific chemical constituents, and biological properties has been described (Figure 1 and Table 1). 

### 5.1. Chondrus crispus

*Chondrus crispus* Stackhouse 1797 is commonly known as the giant Irish moss, jelly moss, and carrageen. The French name of this red alga is mousse d’Irlande. It is an intertidal species and a common inhabitant of rocks near the shores of the northern to middle Atlantic [40]. More information about the plant’s life cycle, history, cell wall biology, reproduction mechanisms, ecological aspects, genes, genomic traits, and commercial uses has been written about elsewhere [41]. *Chondrus crispus*, the most well-studied species of red algae, has garnered significant attention for being used as a good carrageenan source. The polysaccharide carrageenan is usually utilized in the food industry as a thickening agent [34]. A recent transcriptomic study of this algae’s life cycle stages found that genes related to carrageenan-associated cell wall enzymes were differentially expressed [42]. 

Many studies have reported bioactive constituents from different extracts of *C. crispus* using different approaches. The extraction of these compounds involves sample preparation (drying, crushing, milling, and cell wall breakage), followed by extraction techniques including solid liquid extraction, enzyme-assisted extraction, and a few novel extraction methods. Novel approaches include microwave-assisted extraction, ultrasound-assisted extraction (UAE), supercritical-fluid extraction, and pressurized-liquid assisted extraction or high pressure assisted extraction [43]. 

The chemical composition (%) of *C. crispus* as dry matter hydrolysate (1 mg/mL) reported by Kulshreshtha et al. [44] and Adrien et al. [45] consists of protein (27%, 14.4%), neutral sugar (28%, 19.2%), sulfate on the sugar backbone (17%, 9.4%), uronic acids (1.8%, 2.3%), and ash (25%, 13.5%), respectively. In addition, polyphenol content has been reported to be 1.4% [45]. This red alga contains macronutrients (% DW) in the following proportions: carbohydrates (52.6%), protein (10.3–12.4%), and lipids (0.4–5.8%) [46,47,48]. The enzymatic extraction of *C. crispus* using four commercial enzymes, including cellulase, ultaflo, β-glucanase, and proteases (Neutrase) resulted in the presence of monosaccharides (mol%) such as glucose, galactose, and mannose as major fractions and arabinose and xylose as minor fractions [44]. In another study, the major fraction consisted of glucose and galactose (96.4%), while the minor fraction consisted of glucosamine (GlcN, 1.5%), and N-acetylglucosamine (GlcNAc, 2.1%) [45].

Torres et al. [49] reported a protein amount (22.4 mg/g dw) and a total phenolics content (13.4 mg gallic acid equivalents (GAE)/g dw) from the soluble extracts of *C. crispus* obtained by optimizing an UAE approach [49]. Another study documented the presence of flavonoids (202.66 μg/gm) as the major fraction, followed by tannins (13.64 μg/gm) and phenols (12.38 μg/gm) from the methanolic extract of *C. crispus* analyzed using high-pressure liquid chromatography. Among phenolic constituents, catechin (2.335 µg/mL), p-coumaric acid (0.581 µg/mL), and gallic acid (1.09 µg/mL) were found to be major components, while smaller concentrations were observed for protocatechuic acid (0.199 µg/mL), gentisic acid (0.186 µg/mL), p-hydroxybenzoic acid (0.255 µg/mL), and cinnamic acid (0.050 µg/mL) [50]. An earlier study extracted isoflavones from *C. crispus* using the UAE-supercritical fluid approach. Eight isoflavones were recovered, including daidzin, daidzein, genistein, genistin, ononin, sissotrin, formononetin, and biochanin A [51].

The mineral content (mg/100 g DW) of *C. crispus* was found to contain sodium (Na, 4270), K (3184), phosphorus (P, 135), Ca (1120), Mg (732), Fe (17), Zn (7.14), manganese (Mn, 1.32), Cu (<0.5), and I (24.5) [52,53,54]. The presence of β carotene, α-tocopherol, and ascorbate has been reported in *C. crispus* [55,56]. 

Concerning amino acid composition, aqueous ethanolic (75%) extracts of *C. crispus* have been documented to contain common amino acids, including alanine (A), arginine (R), aspartic acid (B), citrulline (Cit), glutamic acid (E), glycine (G), histidine (H), isoleucine (I), leucine (L), serine (S), lysine (K), methionine (M), threonine (T), ornithine, tyrosine (Y), phenylalanine (F), proline (P), taurine, valine (V), and peptides [57]. Another study showed the presence of L-citrullinyl-L-arginin and gigartinine in aqueous extracts of *C. crispus* [58]. 

Research shows that when *C. crispus* is exposed to photosynthetically active radiation or UV radiation, it makes mycosporine-like amino acids (MAAs), which are substances that absorb UV light [59]. MAA compounds have been documented to possess antioxidant and photoprotective functions with potential applications in the cosmetic industry. Algae, fungi, and cyanobacteria lichens have MAA-producing ability [60]. The presence of five different MAAs was recently confirmed in methanolic extracts of wild and cultivated *C. crispus* using the liquid chromatography with tandem mass spectrometry technique (×10^3^) [61]. These are asterina-330, palythine, shinorine, porphyra-334, and palythinol. The study evaluated two wild *C. crispus* samples with high UV and low UV exposure and one cultivated sample. Among all, palythine (19,000), asterina-330 (6160), and shinorine (2440) showed the greatest peak area counts (×10^3^) in cultivated red alga compared to high-UV wild (6930, 4110, and 233) and low-UV wild (5010, 2720, and 268) red alga, respectively. The levels of porphyra-334 showed lower peak counts (×10^3^) in high-UV (24.9) wild red alga and cultivated (23.4) red alga, while a trace amount was observed for low-UV wild alga (8.8). Palythinol was present in trace amounts in wild high-UV and cultivated red alga but not detected in the low-UV wild *C. crispus* [61].

A previous study found a complex lipid pattern in the *C. crispus* extract. The acyl lipid composition of *C. crispus* extract consisted of monoglycosyldiacylglycerol (MGlyDG), diglycosyldiacylglycerol (DGlyDG), sulphoquinovosyldtacylglycerol (SQDG), phatidylcholine (PC), phosphatidylsulphocholine (PSC), phosphatidylglycerol (PG), phosphatidylethanolamine (PE), phosphatidic acid (PA), diphosphatidylglycerol (DPG), triacylglycerol (TAG), free (unesterified) fatty acid (FFA), and esterified sterol (EST). Fatty acid composition (% total acids) of acyl lipids include myristic acid (14:0), palmitic acid (16:0), palmitoleic acid (16:l (*n*-7)), palmitlinoleic acid (16:2), stearic acid (18:0), oleic acid (18:l (*n*-9)), linoleic acid (18:2 (*n*-6)), ɣ-linolenic acid (18:3 (*n*-6)), α-linolenic acid (18:3 (*n*-3)), stearidonic acid (18:4 (*n*-3)), eicosadienoic acid (20:2 (*n*-6)), dihomo ɣ-linolenic acid (DGLA, 20:3 (*n*-6)), arachidonic acid (AA, 20:4 (*n*-6)), eicosapentaenoic acid (EPA, 20:5 (*n*-3)), and others [62]. Notably, PUFAs such as AA and EPA have been documented to be present in very high concentrations in *C. crispus* [63]. Another study documented the presence of AA, EPA, lutein, and eight galactolipids, namely (2S)-1,2-bis-O-eicosapentaenoyl-3-O-β-D-galactopyranosylglycerol, (2S)-1-O-eicosapentaenoyl-2-O-arachidonoyl-3-O-β-D-galactopyranosylglycerol, (2S)-1-O-(6Z,9Z,12Z,15Zoctadecatetranoyl)-2-O-palmitoyl-3-O-β-D-galactopyranosylglycerol, (2S)-1-O-eicosapentaenoyl-2-O-palmitoyl-3-O-β-D-galactopyranosylglycerol, (2S)-1,2-bis-O-arachidonoyl-3-O-β-D-galactopyranosylglycerol, (2S)-1-O-arachidonoyl-2-O-palmitoyl-3-O-β-D-galactopyranosylglycerol, (2S)-1-O-eicosapentaenoyl-2-O-palmitoyl-3-O-(β-D-galactopyranosyl-6-1α-D-galactopyranosyl)-glycerol, and (2S)-1-O-arachidonoyl2-O-palmitoyl-3-O-(β-D-galactopyranosyl-6-1α-D-galactopyranosyl)-glycerol from the methanolic extracts of *C. crispus* [64]. 

For many decades, the use of carrageenans in food applications has been well documented and deemed to be generally recognized as safe (GRAS). Their extraction relies on optimizing numerous parameters, including pH, temperature, process duration, and alkaline pre-treatment, for each type of seaweed. This optimization aims to maximize the seaweed’s structural and gelling qualities. Extended duration and excessive application of alkali agents can inhibit the gelling characteristics and reduce the molecular mass. Additional valuable information regarding carrageenan, including its properties, extraction process, and characterization, can be found in more comprehensive sources [24]. Torres et al. utilized a Box–Behnken design coupled with ultrasound treatment to extract carrageenan from *C. crispus*. This extraction process resulted in carrageenan yields ranging from 26.1 to 43.5 g/100 g dried algae with improved biological properties [49]. 

More recently, Maia et al. [65] optimized a three-step (pigments, proteins, and carrageenan) procedure for bioactive compounds’ extraction from *C. crispus*. Ultrasound-assisted extraction (probe (750 W, 20 kHz) versus bath (120 W, 40 kHz)) was compared for 20 or 40 min and utilized for pigment extraction. The residue from the first step was given an alkaline treatment and the protein component was precipitated using ethanol. The residue obtained from the second phase was subjected to UAE treatment for carrageenan extraction. The study reports the presence of chlorophyll (a) and total carotenoids with mean values varying from 289.2 and 432.2 μg/g extract dw, to 39.9 and 59.9 μg/g dw, respectively. A higher value was reported with the UAE bath device, irrespective of the extraction time. HPLC analysis further identified two carotenoids (fucoxanthin and lutein) and six chlorophyll-a derivatives (pheophythin-A and five unidentified chlorophyll (a) derivatives). UAE extracts contained proteins and carrageenans yields in the ranges of 3.6–41 g/100 g and 29.7–36.1 g/100 g, respectively, while total phenolic content values ranged from 2962 to 3709 μg GAE/g dw [65]. In later sections (Table 1), more information will be discussed about the biological properties of the bioactive components of different extracts of *C. crispus*.

#### 5.1.1. Antimicrobial Activity 

The widespread utilization of antibiotics has had a direct and indirect impact on human existence and on the environment, respectively, which calls for the exploration of natural substances that can regulate the growth of toxin-producing microbes. The use of seaweed extracts as antimicrobial agents has attracted widespread attention [66]. In a similar vein, extracts of the genus *Chondrus* have also been explored as antimicrobials. Chambers et al. [67] documented the anti-fouling property, whereby the ethanolic extracts of dried *C. crispus* were less effective than the fresh seaweed extracts at stopping the growth of phytoplankton and bacterial species [67] (Table 1). Inspired by this study, Salta et al. [68] evaluated the anti-biofilming effects of *C. crispus*. The attachment of *Marinobacter hydrocarbonoclasticus* and *Cobetia marina* was significantly affected at concentrations of 200 ppm and 100 ppm [68]. The extract type may have an effect on the antimicrobial potential of different macroalgae, including *C. crispus*. As per the Cox et al. [69] study, the methanolic extract showed lesser antimicrobial activity, while higher activity was reported with ethanol and acetone as solvents. Notably, methanolic extract enhanced the growth of bacteria, while other solvent extracts inhibited them, especially ethanol extract, which resulted in a 100% inhibition rate against *Enterococcus faecalis* [69]. The study reported minimum inhibitory concentration (MIC) and minimum bactericidal concentration (MBC) values for the selected food spoilage and food pathogenic bacteria (Table 1). 

Another study looked at the antimicrobial activity of *C. crispus* taken from wild and integrated multitrophic aquaculture (IMTA) system regimes using different extraction solvents based on their polarity (diethyl ether, ethyl acetate, methanol, and water). For most of the selected microorganisms (bacteria and yeast species; clinical and food isolates) (Table 1), ethyl acetate extract from the IMTA regime showed the highest inhibition [70]. Recently, the antimicrobial activity of *C. crispus* isolated from Spain was evaluated against different species of Gram-positive and Gram-negative bacteria and yeast species. With minimum fungicidal concentration, MIC, and MBC values, the extract demonstrated antimicrobial activity (Table 1) [71]. The above-mentioned studies show the antimicrobial potential of *C. crispus*. However, synergistic studies that combine different *Chondrus* species with other antimicrobial agents are needed in the near future to develop more effective antimicrobial products. 

#### 5.1.2. Anti-Stress and Immunomodulation Activity

Stress is a body’s response to challenging circumstances that might disturb homeostasis due to various intrinsic and extrinsic factors [72]. Studies have shown that stress can dysregulate the immune responses of the body [73]. Natural compounds from terrestrial and aquatic flora and fauna and bacterial and fungal species are being researched against stress, aging, and related diseases [74]. Investigations on red algal species are also underway. In this direction, a study demonstrated the stress-relieving potential of the methanolic extract of *C. crispus* in the juglone-induced stress model of a soil nematode, *Caenorhabditis elegans* [75]. Juglone (5-hydroxy-1,4-naphthoquinone) is a plant-derived compound that can be used as an ROS elicitor and herbicide and has been found to be toxic for marine organisms [76,77]. *Caenorhabditis elegans* is known to mimic the human genome and physiological responses, making it suitable to check the effects of natural products [78]. The extract reduced oxidative stress and enhanced life span by upregulating expressions of stress-resistance genes (Table 1). The study also assessed the presence of bioactive constituents in the extract and found that pigments, unsaturated fatty acids, and glycolipids led to stress tolerance in worms [75]. 

Another study found that adding *C. crispus* water extract (CCWE) and its components (Kappa-carrageenan (K-CGN)) to the diet of nematodes made their immune systems stronger against a clinical isolate of *Pseudomonas aeruginosa*. Extract supplementation led to an increased expression of innate immune genes in the nematode. Both CCWE and K-CGN extended the life span, increased reproduction, decreased aging, and improved the overall physiology of the worms. The authors highlighted the importance of Kappa-carrageenan present in the extract in enhancing the immune response [79] (Table 1). These studies indicate the protective effects of *Chondrus* in stressful conditions. More research is needed using cell and animal models in the near future.

#### 5.1.3. Nitric Oxide Inhibition

Nitric oxide (NO) is an essential signaling molecule that has been documented to control many physiological processes. However, its excessive generation from L-arginine catalyzed by nitric oxide synthases can lead to complications in host tissue [80]. 

Banskota et al. [64] showed the NO-inhibition potential of a methanolic extract of *C. crispus*. At 100 µg/mL concentration, the extract was demonstrated to have 15.6% NO inhibition. Further, after separating the extract into organic and water fractions, the organic extract showed enhanced 64.6% NO-inhibition, while no activity was observed for the water extract. The organic fraction underwent three additional sub-fractions, and the identified compounds showed dose-dependent NO-inhibition activity (Table 1). This study reports that compounds from methanolic extract have the potential to deal with NO-stimulated inflammatory disorders [64].

#### 5.1.4. Neuroprotection

Alongside other health-promoting activities, the bioactive constituents from macroalgae have become increasingly popular due to their neuroprotective properties, making them intriguing options for the protection and treatment of many neurodegenerative diseases [81]. The diagnosis of Alzheimer’s disease (AD) requires the presence of two distinct pathological features: extracellular plaque deposits of the β-amyloid peptide (Aβ) and neurofibrillary tangles composed of the microtubule binding tau protein. As described in the earlier section, the methanolic extract of *C. crispus* showed anti-stress activity in *C. elegans*. The same research group established the neuroprotective role of the methanolic extract of *C. crispus* using the transgenic *C. elegans* as a model organism. The research showed that certain parts of seaweed, especially glycolipids, slowed down the paralysis caused by beta (β)-amyloid treatment. Extract supplementation also lowered the amount of Aβ peptide that built up in the worm and increased the expression of several stress-relieving genes (Table 1). This eventually decreased the levels of reactive oxygen species (ROS) in the supplemented transgenic nematode. The authors suggested ROS levels were mitigated following the activation of the skinhead 1 (SKN1, functional analogue of nuclear factor erythroid 2-related factor 2) pathway. The study also found that the extract’s effect on the Aβ peptide was a post-transcriptional one, since immunoblotting showed that it blocked the protein [82]. The study indicates the potential neuroprotective properties of this red seaweed in a nematode model. Future studies should focus on cell and animal models for neurodegenerative disorders. 

#### 5.1.5. Improvement of Gut Health

Prebiotics are non-digestible food constituents that selectively enrich the beneficial microbiota in the colon microbiota. They also act as raw materials for the generation of biologically active substances [83,84]. There is a growing body of research focused on investigating the health benefits of polysaccharides found in seaweeds. In one such study, Liu et al. demonstrated the prebiotic effect of cultivated red seaweed extract (basal diet + 0.5% and 2.5% (dry *w*/*w*) *C. crispus*) on male Sprague–Dawley rats. The extract supplementation improved gut health in treated animals [85]. No effect of *C. crispus* extracts or fructo oligosaccharide treatments (0.5% and 2.5%, standard) on body and organ weights was observed, while plasma levels of immunoglobulins (IgG and IgA) were elevated with the lower dose of *C. crispus* (0.5%). Phylochip array analysis results showed that red seaweed increased abundance of beneficial colonic microbiota with a parallel reduction in pathogenic microbes (Table 1). In particular, adding 2.5% *C. crispus* increased the amount of *Bifidobacterium breve* and decreased the amount of pathogenic bacteria like *Streptococcus pneumonia* and *Clostridium septicum*. Short-chain fatty acids (SCFAs) like propionic, acetic, and butyric acids were found in higher amounts in the poop of rats that had been treated [85]. The study indicates the potential of *C. crispus* as a functional food owing to its multiple prebiotic effects.

#### 5.1.6. Antiproliferative Activity 

Cancer is one of the deadliest diseases, leading to an increased number of deaths worldwide [86]. Over the years, many natural compounds have been documented to have anticancer effects [87]. Recently, marine natural compounds have also been investigated as potential anticancer agents [12]. Though limited studies have been documented on the anticancer ability of the genus *Chondrus*, the genus has been documented to withstand oxidative stress resulting from fluctuations in different climatic conditions. This ability to endure can be attributed to the presence of cellular constituents (Ascorbate, MAAs, and α-tocopherol) in alga [55,56,59], which may aid in the biological properties of C*hondrus*. In a study by Athukorala et al. [61] antiproliferative effects of the wild (harvested) and cultivated extracts of *C. crispus* were observed against the human adenocarcinoma cervical cell line (HeLa) and histiocytic lymphoma cell line (U-937). Both wild and cultivated extracts showed the same MAA profile; however, cultivated *Chondrus* species exhibited significant apoptotic morphological changes (shrinkage and rounding) in HeLa cells. It was much better at getting rid of free radicals (mg ascorbic acid equivalents/g extract) and oxygen radicals (µmoles trolox equivalents/g extract) than the wild extracts (Table 1) [61]. The authors advocated that the instigation of apoptosis was responsible for the antiproliferative activity [61]. 

A recent study reported the cytotoxic behavior of methanolic extracts of dried algal powder of *C. crispus* against the hepatic tumor cell line (HepG2), breast cancer cells (MCF7), colorectal adenocarcinoma (Caco-2), and adenocarcinoma of human alveolar cells (A549). Among all of them, algal extract killed (cytotoxicity) a lot of cells, 81.9% of HepG2 and 71.8% of A549 cells compared to the reference drug sorafinib, which killed only 69% and 71% of cells, respectively [50] (Table 1). 

Carrageenan fraction generated by the optimal UAE approach demonstrated antitumoral potential by inhibiting ovarian carcinoma cells (A2780, 96%), colon carcinoma cells (HT29, 95%), lung carcinoma cells (A549, 94%), and cervix carcinoma cells (HeLa 229, 91%) in human cancer cell lines [49]. There are different bioactive components in wild and cultivated species of *C. crispus* that make it very good at fighting cancer. However, more in vitro and in vivo studies are needed to fully utilize this genus’s potential.

#### 5.1.7. Anti-Oxidant Activity 

A significant factor in the onset of many chronic diseases is oxidative stress, which results from the production of free radicals. Free radicals include reactive oxygen species (ROS) and reactive nitrogen species. They can change the redox status and cause oxidative stress in many macromolecules [88]. Macroalgal extracts are considered to be an important source of natural compounds with antioxidant potential. Examples include phenolic compounds, terpenoids, meroterpenoids, nitrogenous compounds, and carbohydrates and polysaccharides [89].

Recently, the methanolic extract of *C. crispus* demonstrated a dose-dependent anti-radical activity and induced inhibition of 2,2′-azino-bis (3-ethylbenzothiazoline-6 sulfonic acid (ABTS) and 2,2-diphenyl-1-picryl-hydrazyl-hydrate (DPPH) by 120.29% and 84.17%, respectively, compared to the Trolox standard (100.25%) and butylated hydroxytoluene standard (91.51%) at the highest concentration (200 μg/mL). Also, the total antioxidant activity of the *C. crispus* extract (μg) changed with dose. It was 235.81 ± 3.20 at the highest concentration (400 μg), about the same as the vitamin C control (243.46 ± 3.56) [50] (Table 1). More recently, a UAE approach (probe and bath) operated at different time spans (20 and 40 min) showed ABTS and DPPH scavenging values of 1530 and 2145 μg TE/g extract dry weight (dw) and 982 and 2058 μg TE/g extract, respectively. The UAE bath (20 min) displayed lower scavenging activity for both assays. The study also reported EC_50_ except for the DPPH 20 min approach (Table 1). Inhibition of superoxide radical anion was observed only at bath approach for both time spans (Table 1) [65]. In another study, the ABTS radical scavenging (maximum) ability of *C. crispus* soluble extracts was found to be 182.4 mg TE/g dw [49]. 

#### 5.1.8. Antiviral Activity

Regarding antimicrobial action, numerous medicines have specifically been targeted towards bacterial infections due to a deeper understanding of the molecular mechanisms underlying bacterial pathogenesis. However, there are a limited number of antiviral drugs that have been deemed safe for human use, mostly because the viral pathogenesis remains uncertain [90]. No doubt, the available drugs are effective against the herpes virus; however, the development of drug resistance and adverse effects on human health necessitate the development of novel anti-herpetic agents. On this line, enzyme hydrolysates of *C. crispus* extract showed significant activity against the *Herpes simplex virus*, with an EC_50_ value ranging from 77.6 μg/mL to 126.8 μg/mL. Red seaweed extraction using commercial proteases (P1) and carbohydrases (C1, C2, and C3) showed a higher dry biomass yield compared to an aqueous extraction procedure. Better results were seen with fractions made with C1, C3, and P1. This might be because polysaccharides contain sulfate [44] (Table 1). 

Recently, a randomized, placebo-controlled study was conducted on 394 individuals for 21 days to assess the preventing effect of a nasal spray containing ι-carrageenan (0.17%) against COVID-19 disease in healthcare workers in Argentina. The incidence of COVID-19 differed significantly between the treatment and placebo groups. The relative risk reduction was 79.8% (95% CI 5.3 to 95.4; *p* = 0.03), and the absolute risk reduction was found to be 4% (95% CI 0.6 to 7.4). The study reported one adverse effect (*p* = 0.5) in the treatment (17.3%) and placebo (15.2%) groups. The investigation suggests the significant protective effects of a nasal spray supplemented with ι-carrageenan in healthcare workers against COVID-19 [91]. The research findings indicate the anti-viral properties of polysaccharides from *Chondrus* sp. More such studies are warranted in the near future against other deadly viruses as well.

#### 5.1.9. Anti-Coagulation Activity

Blood coagulation is a vital defense mechanism for preventing bleeding and life-threatening hemorrhagic complications. Generally, the blood coagulation process involves two pathways, intrinsic and extrinsic, with a cascade of reactions that involves many factors. Both of these pathways merge into a common pathway. The anticoagulant activity is usually determined using the following assays: prolongation of activated partial thromboplastin time (APTT), thrombin time (TT), and prothrombin time (PT) assays. Heparin and heparin-derived anticoagulants are accessible but not without side effects such as bleeding risk and other complications [92,93]. Therefore, the quest is still ongoing to look for natural anticoagulants with fewer side effects. The plausibility of marine life has also been explored in this direction [93]. 

Researchers have explored the potential of *Chondrus* extract as an anticoagulant. The crude aqueous extract’s ability to stop blood clots was first tested against the clotting process using various anticoagulant pathways, such as the intrinsic and/or common (APTT), the specific antithrombin-dependent pathway (anti-Xa and anti-IIa), the common (TT), and the extrinsic (PT). Among all, *C. crispus* showed APTT activity, revealing they are mostly effective against intrinsic/or common pathways (Table 1). The study compared the results to those of heparin and Lovenox^®^ standards and shows that *C. crispus* crude extract may act as anticoagulant [45]. More studies are required for other *Chondrus* species and purified extracts to be assessed as potential anti-coagulants.

#### 5.1.10. Antivenom Activity

Snakebite envenomations represent a significant and overlooked communal health concern on a global scale, particularly in tropical and subtropical areas of developing nations [94]. Antivenom is accessible, but not without adverse reactions. As a result, other approaches, such as the utilization of plant-derived venom-detoxifying substances are gaining popularity [95]. Recent research has also indicated the use of algal species as potential venom neutralizing agents [96]. In red seaweeds, the antivenom activity of *C. crispus* was evaluated against the venoms of snake species, either *Bothrops jararaca* or *B. jararacussu*. The research used lambda (λ) carrageenan and tested it against venom toxicity both in the lab and on animals (Table 1). It was revealed that the extract supplementation lessened the harmful effects of bleeding (hemorrhage), swelling, muscle damage, and death, both before and after the venom injection, irrespective of the mode of administration. Further, gel formulations using *C. crispus* λ carrageenan were also found to be effective against hemorrhage following venom injections [97]. The study suggests the potential anti-venom property of the polysaccharide from *C. crispus*. Especially, the concept of topical application of *C. crispus* λ carrageenan in a gel form could be a beneficial approach for snake bite victims in the near future.

**Table 1:** Health-promoting effects of different species of the red alga genus, *Chondrus*.

Species	Extract Type	Target Models	Biological Activities	Ref.
**Antimicrobial activity**				
*C. crispus*	Crude ethanol (95%) Dried and fresh extracts	Marine bacterial species: *Pseudoalteromonas elyakovii*, *Polaribacter irgensii*, *Vibrio aestuarianus*, *Shewanella putrefaciens*, and *Halomonas marina* Microalgae species: *Chlorarachnion reptans*, *Chlorarachnion globosum*, *Exanthemachrysis gayraliae*, *Cylindrotheca cloisterium*, *Navicula jeffreyi*	Potential antifoul activity Dried extracts had lower MIC than fresh extract. MIC (μg/mL) dried; fresh: *P. elyakovii* (10; 25), *P. irgensii* (10; 25), *V. aestuarianus*, (25; 50), *S. putrefaciens*, (25; 25), and *H. marina* (10; >50); *C. reptans* (10; 25), *C. globosum* (25; 10), *E. gayraliae* (10; 10), *C. cloisterium* (1; 10), and *N. jeffreyi* (25; 25).	[67]
*C. crispus*	Crude ethanolic extract of dried algae	*Cobetia marina* (50–200 ppm), *Marinobacter hydrocarbonoclasticus* (100–200 ppm)	Anti-biofilming effect	[68]
*C.crispus*	Methanol, ethanol and acetone (60%)	*L. monocytogenes*, *P. aeruginosa*, *Salmonella abony*, and *E. faecalis*	(%inhibition) (methanol; ethanol; acetone): *L. monocytogenes* (−3.88; 50.27; 56.13), *S. abonya* (−10.70; 0.80; 4.70), *E. faecalis* (−66.08; 100.00 ± 0.38; −89.74), *P. aeruginosa* (−31.72; 61.51; 81.74)	[69]
*C. crispus*	Ethyl acetate	*S. enteridis*, *E. coli*, *P. aeruginosa*, *L. innocua*, *B. cereus*, *S. aureus*, *L. brevis. E. faecalis*, *Candida* sp.from both wild and IMTA regimes	Ethyl acetate: inhibitory activity in both regimes; best in IMTA	[70]
	Diethyl ether		Diethylether extract: (1) IMTA regime: no effect against *S. aureus* (CI and FI), *L. brevis* and *E. faecalis* (2) wild regime: no effect against *S. enteritidis*	
	Methanol:Water (1:1)		Methanol:Water extract: IMTA: no effect against *Candida* sp. Wild: no effect against *S. enteritidis* and *S. aureus* (CI)	
*C. crispus*	Acidified ethanol	Bacterial species: *Bacillus cereus*, *Micrococcus flavus*, and *Staphylococcus aureus*, *Proteus mirabilis*, *Salmonella* Typhimurium Yeast species: *Candida albicans C. tropicalis*, and *C. krusei*	Anti-bacterial (mg/mL) (MIC, MBC): *B. cereus* (0.045, 0.06), *M. flavus* (0.09, 0.12), *S. aureus* (0.06, 0.1), *P. mirabilis* (0.045, 0.06), and *S.* Typhimurium (0.06, 0.12). Anti-fungal (mg/mL) (MIC, MFC): *C. albicans* (0.045, 0.06), *C. tropicalis* (0.045, 0.06), and *C. krusei* (0.06, 0.12).	[71]
**Anti-stress and immunomodulation activity**				
*C. crispus*	Methanol extract (CCME)	Juglone (300 or 500 μmol)-induced *Caenorhabditis elegans +* CCME (1 mg/mL)	Anti-stress activity ↓ROS, stress response genes: ↑*sod3*, ↑*hsp16.2*, ↑*skn1*, ↑transcription factor daf16	[75]
*C. crispus*	Aqueous CCWE and K-CGN	*Caenorhabditis elegans* + infection with *Pseudomonas aeruginosa* PA14 CCWE (0, 250, 500, or 750 μg/mL) or K-CGN (200 μg/mL)	↑Survival rates CCWE = 500 μg/mL (optimum dose) and K-CGN Immune gene expression: ↑*hsf-1*, ↑*irg-1*, ↑*irg-2*, ↑*F56D6.2*, ↑*F49F1.6*, ↑*K05D8.5*, ↑*C29F3.7*, ↑*ZK6.7*, ↑*abf-1* ↑*F28D1.3*, ↑*F38A1.5*, ↑*lys-1*, and ↑*spp-1*. Repressed QS (lasI/R and rhlI/R) systems. Virulene factor genes: ↓*sbe*, ↓*hcnC*, ↓*aroE*, ↓*rpoN*, and ↓*sodB*. Inhibited biofilm formation	[79]
**Nitric oxide inhibition**				
*C. crispus*	Methanol extract of cultivated alga	RAW264.7 cells + LPS (1 µg/mL), extract (25, 50, and 100 µg/mL), 24 h	Dose-dependent NO inhibition	[64]
**Neuroprotection**				
*C. crispus*	Methanol extract; organic fraction	*C. elegans* CL4176 + β amyloid toxicity organic fraction (0.1–1.0 mg/mL)	↓amyloid species deposition ↑antioxidant activity Stress response genes: ↑*sod3*, ↑*hsp16.2*, and ↑*skn1*	[82]
**Improvement of gut health**				
*C. crispus*	Cultivated alga and FOS	Male Sprague Dawley rats (1) Basal diet (control) (2) the basal diet + 2.5% or 0.5% (dry *w*/*w*) of cultivated seaweed (3) basal diet + 2.5% or 0.5% of FOS (dry *w*/*w*)	No change in body and organs weights Cultivated alga extract (0.5%): ↑IgA, ↑IgG Alga extract (2.5%): Modulated gut microbiota ↑*Bifidobacterium*, ↑*Legionella*, ↑*Sutterella*, ↑*Blautia*, ↑*Holdemania*, ↑*Shewanella*, ↑*Agarivorans* and ↓*Streptococcus* ↑SCFAs	[85]
**Antiproliferative activity**				
*C. crispus*	Methanolic extract Wild (high-UV and low-UV) and cultivated	HeLa cells and U-937 cells: 0.125, 0.5, 1,2, and 4 mg/mL, 24 h	Showed antiproliferative effect on HeLa cells: (upto 1 mg/mL): wild low-UV > wild high-UV > cultivated. (at 2 and 4 mg/mL) cultivated > wild low-UV > wild high-UV. U-937 cells: (upto 1 mg/mL): wild high-UV > wild low-UV > cultivated. Cutlivated extract in HeLa cells: ↑caspase-3,↑caspase-7; cell cycle arrest at Sub G1 (apoptotic) Antioxidant: reducing ability: cultivated > wild low-UV > wild high-UV ORAC: cultivated > wild low-UV > wild high-UV	[61]
*C. crispus*	Dried algal powder, 80% methanol	HepG2 cells, MCF7 cells, Caco-2 cells, and A549 cells, (0.01, 0.1, 1.0, 10, and 100 µg/mL), 24 h control (Sorafenib)	% inhibition (IC_50_ µg/mL): HepG2, control (1.32, 2.23); MCF7 cells, control (179, 4.0); Caco-2 cells, control (8.24, 2.88); A549 cells, control (7.90, 2.55)	[50]
*C. crispus*	Carrageenen fraction	A-2780, A-549, HT-29, and Hela-229 (0.1 mg/mL)	IC_50_ (mg/mL) = A-2780 (0.0080); A-549 (0.0099); HT-29 (0.0211); Hela-229 (0.0492)	[49]
**Antioxidant activity**				
*C. crispus*	Dried algal powder 80% methanol	DPPH and ABTS assay: at 50, 100, 150, and 200 μg/mL TAC: at 100, 200, 300, and 400 μg/mL	DPPH (% inhibition): 50 μg/mL (25.0), 100 μg/mL (37.8), 150 μg/mL (47.3), and 200 μg/mL (84.2), BHT (μg) 91.5 ABTS (% inhibition): 50 μg/mL (82.0), 100 μg/mL (98.0), 150 μg/mL (108.9), and 200 μg/mL (120.3), Trolox (μg) (100.2). TAC: 100 μg/mL (88.48), 200 μg/mL (94.77), 300 μg/mL (136.88), and 400 μg/mL (235.81), vitamin C (μg) 243.46	[50]
*C. crispus*	Extract with UAE treatments	DPPH and ABTS @ 4 treatments (A) Probe 20 min; (B) Probe 40 min; (C) Bath 20 min; (D) Bath 40 min. Superoxide radical @ 2 treatments anion (C) and (D), 0.5, 1, and 2 mg/mL)	EC_50_ (mg/mL) DPPH: (A) ND, (B) 6.3, (C) ND, (D) 7.1 EC_50_ (mg/mL) ABTS: (A) 3.1, (B) 2.4, (C) 4.6, (D) 2.5 % inhibition (2 mg/mL): (C) 21.1 (D) 27.3	[65]
*C. crispus*	UAE extracted soluble extract	ABTS (1 mL) + 10 mL (soluble extract) or Trolox	Trolox equivalent antioxidant capacity (TEAC) = 182.4 mg/g	[49]
**Antiviral activity**				
*C. crispus*	Enzymatic hydrolysates (P1, C1, C2, and C3)	African green monkey kidney cell line and HSV-1 (wild-type strain 17), hydrolysates (1–200 μg/mL (50 μL))	EC_50_ (μg/mL): 77.6 –126.8 μg/mL P1 (neutrase) = 77.6; C1 (cellulase) = 103.3; C2 (β-glucanase) = 126.8; C3 (ultaflo) = 109.3	[44]
**Anticoagulation activity**				
*C. crispus*	Crude aqueous extract	0.125, 1.25, 12.5, and 125 μg/mL	Residual Xa activity * = *C. crispus*: 253; Heparin: 0.20; Lovenox^®^: 0.15; Residual IIa activity * = *C. crispus*: 194; Heparin: 0.25; Lovenox^®^: 1.8; APTT ** = *C. crispus*: 21; Heparin: 0.7; Lovenox^®^: 4.5; PT ** = *C. crispus*: >415; Heparin: 16; Lovenox^®^: 495; TT ** = *C. crispus*: <125; Heparin: 0.45; Lovenox^®^: 1.82	[45]
**Antivenom activity**				
*C. crispus*	Lambda-carrageenan	In vitro assays Hemolytic/proteolytic activity: human erythrocytes and egg yolk emulsion (substrate) *B. jararaca* or *B. jararacussu* venom (20 μg/mL) + polysaccharide (200 μg/mL), 30 min at 25 °C Coagulation: venoms (10 μg/mL), + polysaccharide (200 μg/mL)	Inhibited hemolytic activity (60%) for *B. jararaca* Inhibited proteolytic activity: *B. jararaca* (65%) *B. jararacussu* (100%) Impaired plasma coagulation	[97]
		Balb/c mice Antihemorrhagic activity (a) Inhibtion protocol: incubation—polysaccharide (150 μg/mouse) or antivenon was mixed with 2 MHD of venom of *B. jararacussu* (50 μg/mouse) or *B. jararaca* (30 μg/mouse), 30 min, 25 °C. S.C. route (b) Treatment protocol: venoms (30 μg/mL, S.C.) + λ carrageenan 30 min later (S.C., samesite), or I.V. (c)venoms injection (S.C.), 30 min later (one injection) or 30 + 30 min (two injections) of polysaccharide (I.V.)	Inhibition protocol *C. crispus* polysaccharide inhibited venom’s hemorrhage: *B. jararaca* (40%) and *B. jararacussu* (100%). Treatment protocol *C. crispus* polysaccharide inhibited venom’s hemorrhage: *B. jararaca* (20%) and *B. jararacussu* (40%). *C. crispus* polysaccharide (one injection: two injections) inhibited venom’s hemorrhage: *B. jararaca* (20%:50%) and *B. jararacussu* (40%:60%).	
		Antihemorrhagic activity of gel (a) Prevention protocol: Venoms dose (25 μg/mouse) (S.C.), + polysaccharide gel (100 μg) topically applied 15 or 30 min later, + antivenom single injection (I.V.) (b) Treatment protocol: gel containing λ polysaccharide (100 μg/mL) topical application + venoms (S.C., 25 μg/mouse) 15 or 30 min later.	Inhibition of hemorrhage after topical application of polysaccharide based gel, irrespective of protocol Hemorrhage of *B. jararacussu* was inhibited fully in a 30 min topical application before venom injection. Hemorrhage of *B. jararacussu* inhibited 30% in a 15 min topical application before venom injection	
		Antidematogenic activity single, sub-plantar injection into the right paw (50 μL), after 1 h, the paw’s amputation. λ carrageenan (150 μg/mouse) or antivenom + venoms (25 μg/mouse), 30 min at 25 °C, injected (50 μL of the mixture) (S.C.) Protocol of treatment: venoms (S.C.), λ polysaccharide or venom 15 or 30 min later, (I.V.)	Inhibition (%) of edema (~30%) λ polysaccharide mixed with venom inhibited edema (45%) λ polysaccharide is more effective against *B. jararacussu* than *B. jararaca*	
		Antilethal activity Protocol of incubation: venoms (130 μg/mouse) + λ polysaccharide (100 μg/mouse) or antivenom, 30 min at 25 °C (I.P. injection) Protocol of treatment: I.P. injection of venoms, 30 min later, λ polysaccharide or antivenon (I.V.).	Polysaccharide + venom protected the mice (both treatments)	
		Antimyotoxic activity Protocol of incubation: *B. jararacussu* venom (50 μg/mouse) + polysaccharide (150 μg/mouse), saline or antivenon, 30 min at 25 °C, 100 μL injection. Protocol of treatment: *B. jararacussu* venom (I.M.), polysaccharide, antivenom, or polysaccharide + antivenom, 30 min later (I.V.)	Inhibited myotoxic activity. Slight protection was observed.	
**Antitumor and immunomodulation**				
*C. ocellatus*	EA polysaccharides: PC1, PC2, PC3, PC4, and PC5	ICR mice, S180 and H22 tumor cells (subcutaneous implatation), 0.2 mL of each extract (200 mg/kg/day) for 7 days	Inhibited tumor growth (%) PC1, PC2, PC3, PC4, and PC5 S180: (57.58,37.64, 44.35,50.52, and 66.15) H22: (57.03,61.90, 23.22,68.97, and56.90) ↑spleen weights, ↑NK cell activity, ↑lymphocyte proliferation	[98]
*C. ocellatus*	Polysaccharide PC5	ICR mice, S180 (subcutaneous implatation) (1) 5-FU:25 mg/kg (2) PFp1 PC5:100 mg/kg (3) PFp2 PC5:50 mg/kg (4) PF1 PC5+5-FU:100 + 25 mg/kg (5) PF2 PC5+5-FU 50 + 25 mg/kg	Inhibited tumor growth (%): 5-FU: (37.30), PFp1: PC5 (32.08), PFp2: PC5 (26.03), PF1: PC5+5-FU (63.87), PF2: PC5+5-FU (55.40) ↑TNFα ↑lymphocyte proliferation ↑spleen weights	[99]
*C. ocellatus*	Polysaccharide PC4	ICR mice, H22 (subcutaneous implatation) (1) 5-FU:25 mg/kg (2) HFp1 PC4:100 mg/kg (3) HFp2 PC4:50 mg/kg (4) HF1 PC4+5-FU:100 + 25 mg/kg (5) HF2 PC4+5-FU 50 + 25 mg/kg	Inhibited tumor growth (%) 5-FU: (30.76), HFp1 PC4: (43.97), HFp2 PC4: (35.37), HF1 PC4+5-FU: (51.73), HF2 PC4 + 5-FU (47.01) ↑lymphocyte proliferation ↑TNFα ↑spleen weights	[100]
**Anti-inflammatory activity**				
*C. ocellatus* Holmes	Ethanol extract (COHEE)	LPS +RAW 264.7 cells, (0.1, 1, 10, 50, and 100 μg/mL), 22 h	No cytotoxic effects ↓NO, ↓IL-6, ↓TNF-α, ↓IL-1β, ↓iNOS, ↓COX-2, ↓NF-κB p65, ↓p-MAPK	[101]
		Croton oil-induced mouse ear edema model, COHEE (10, 50, and 250 mg/kg BW), 200 μL, croton oil (2.5%, 20 μL/ear), 1 h before treatment	↓Mouse ear edema at a higher dose ↓Mast cell number	
**Antidiabetic activity**				
*C. ocellatus*	Ethanol extract (61%)	In vitro assay	Anti-radical activity: DPPH = (13.11–25.77%), ABTS = (43.48–53.50%) H_2_O_2_ inhibition (IC_50_) = 18.4–85.6 U/mL α-glycosidase inhibition: IC_50_ = 16.92 mg/mL α-glucosidase inhibition: 375.3 mg/mL	[102]
**Cytoprotective and antimicrobial activity**				
*Mazzaella canaliculata*	Methanol extract	In vitro assays	DPPH radical scavenging: IC_50_ = 0.25 mg/mL Antimicrobial activity: *Salmonella* Typhimurium, *Klebsiella pneumoniae*, *Listeria monocytogenes*, *Actinomyces* sp., *Enterococcus faecalis*, *Enterobacter* sp., and *Micrococcus luteus*	[103]
		Wistar female rats (1) Non-treated rats (positive control). (2) maneb (MB I.P.)/kg (300 mg). (3) 300 mg/kg of MB (I.P.) + algal extract (150 mg/kg, oral) (4) algal extract (150 mg/kg), positive control group, 7 days	MB-treated group: ↓body weight, ↓RBC, ↓WBC, ↓viability, ↑DNA damage, ↑platelet rates Co-treated: ↑body weight, ↑RBC, ↑WBC, ↑viability; ↓DNA damage, lowered platelet rates compared to the MB-treated group. Improved mineral levels in blood, bone, and urine In co-treated erythrocyte and bone: ↓MDA, ↓AOPP, ↑SOD, ↑GSH, and ↑GPx	
*Mazzaella canaliculata*	Polysaccharide (CCP)	2, 4, 6, 8, 10, and 12 mg/mL	DPPH (2 mg/mL) has strong antiradical activity comparable to gallic acid. Protective effect against β-Carotene bleaching inhibition assay (10 mg/mL = 39.90%) Ferrous ion chelation: 10 mg/mL = 96.37% Ferric-reducing activity: 10 mg/mL = 2.16 Protection against hydroxyl radical-induced DNA damage	[104]
		Wistar female rats Group 1: control group (saline) Group 2: MB (300 mg/kg, I.P.) Group 3: MB (300 mg/kg, I.P.) + CCP (100 mg/kg), Group 4: MB (300 mg/kg, I.P.) + CCP (200 mg/kg), Group 5: CCP (100 mg/kg, I.P.) (positive control) Group 6: CCP (200 mg/kg, I.P.) (positive control)	Dose-dependent significant improvement in MB’s oxidative and histological injuries. In plasma: ↓urea, ↓creatinine, ↓albumin Co-treatment: ↓MDA, ↓AOPP, ↑SOD, ↑GSH, and ↑GPx Co-treatment: ↑RBCs, ↑WBCs, ↑iron, ↑MCV, ↑MCH and ↑MCHC ↓apoptosis	
**Anti-atopic activity**				
*Mazzaella canaliculata*	Ethanolic extract (CCEE)	BALB/c mouse 1% (*w*/*v*) DNCB, 3 times a day; after 1 week, apply 0.3% (*w*/*v*) DNCB to the same area once a day (200 μL) DNCB + CCEE	↓IFNγ, ↓IL-4 ↓clinical severity score	[105]
**Anti-inflammatory activity**				
*C. armatus*	LMW and HMW carrageenan	Acetic acid-induced colitis in male Swiss mice + oral pretreatment (carrageenan in H_2_O) (5, 10, 50 mg/kg)	HMW: ↓ colon damage, ↓MPO. Effective dose: 5 mg/kg LMW: no protective effect.	[106]
**Antitumor and immunomodulation activity**				
*C. armatus*	Native κ- and λ-carrageenans LMW κ- and λ-carrageenans degradation products	FLO1, KYSE30, and human dermal fibroblast cell lines Treatments: 50, 100, and 400 μg/mL, 24 or 48 hr PBMC + test polysaccharides (1, 10, or 100 μg/mL) or LPS (0.1 μg/mL), 24 h	↓ FLO1 and KYSE30 viability. All polysaccharides showed anti-metabolic activity. In FLO1: LMW κ- and λ-carrageenan were more effective (at 400 μg/mL): LMW κ (%): 48; HMW κ (%): 97.7. LMW λ (%) 61.9; HMW λ (%) 79.1. In KYSE30, naïve κ- and λ-carrageenans were more effective: κ-carrageenans (%) 47.5;. λ-carrageenans (%) 55.1. All carrageenans: induce monocytes to produce cytokines: IL1β, IL6, IL18, and TNFα. LMW λ-carrageenan only: IL10	[107]
*C. armatus*	κ- and λ-carrageenans Native or HMW. LMWDPs	In vitro: murine peritoneal macrophages Control, LPS, HMW-κ, LMWDPs-k, HMW-λ, LMWDPs-λ (1, 10, and 100 μg/mL) or LPS (0.1 μg/mL)	↓phagocytic activity by molecular weight and chemical structure-dependent manner. Anti-phagocytic efficacy = κ-carrageenan > λ-carrageenan At 100 μg/mL: LMWDPs-κ, HMW- λ, no effect on phagocytosis. HMW-κ reduced by 34%	[108]
		In vivo: male C57BL/6 mice Control, LPS, stress, HMW-κ and λ, LMWDPs-κ and λ, 100 μg/kg/day, 7 days	No significant change in body weight or internal organs. Total leucocyte counts = not affected except for κ-carrageenan. Cell motility: LMWDPs (κ): no effect; LMWDPs (λ): 24% reduction. HMW (λ): ↑peritoneal macrophages (40%).	
*C. armatus*	κ- and λ-carrageenan	KYSE-30, FLO-1, HCT-116, RKO, and RPE-1 cell lines	Cytotoxicity (IC_50_ values) of κ- and λ-carrageenan: KYSE-30: 394, 392; FLO-1: 405, 184; HCT-116: 347, 206; RKO: 350.6, 248.3; RPE-1: 728, 615. Delayed cell cycle at different stages. λ-carrageenan in RKO: ↓CDK2, ↓E2F2, ↓cyclin E. Induction of apoptosis	[109]

MIC: minimum inhibitory concentration; IMTA: integrated multitrophic aquaculture system; CI: clinical isolate; FI: food isolate; MBC: minimum bactericidal concentration; MFC: minimum fungicidal concentration; CCWE: *Chondrus crispus* water extract; ROS: reactive oxygen species; sod3: superoxide dismutase 3; hsp16.2: heat shock protein 16.2; skn1: skin head 1; K-CGN: kappa-water-soluble polysaccharide carrageenan; hsf-1: heat shock factor1; irg-1/2: infection response gene 1/2; F56D6.2: C-type lectin; F49F1.6: ShK domain-like, secreted surface protein; K08D8.5/C29F3.7: CUB-like domain; ZK6.7: lypase; abf-1: antibacterial protein; F28D1.3: thaumatinlike protein; F38A1.5: lectin family protein; lys-1: lysozyme-like protein; spp-1: saponin-like protein; QS: quroum sensing; LPS: lipopolysaccharide; NO: nitric oxide; FOS: fructooligosaccharides; IgA: immunoglobulin A; IgG: immunoglobulin G; SCFAs: short -chain fatty acids; UV: ultra violet; ORAC: oxygen radical absorbance capacity; IC_50_: half maximal inhibitory concentration; DPPH: 2,2-diphenyl-1-picrylhydrazyl; ABTS: 2,2-azino-bis-3-ethylbenzothiazoline-6-sulphonic acid; TAC: total antioxidant capacity; BHT: butylated hydroxy toluene; UAE: ultrasound-assisted extraction; ND: not defined; P: proteases; C1/2/3: carbohydrases; HSV-1: *Herpes simplex virus*-1; EC_50_: half maximal effective concentration; APTT: activated partial thromboplastin time; PT: prothrombin time; TT: thrombin time; SC: subcutaneous; IP: intraperitoneal; IV: intravenous; IM: intramuscular; λ: lambda; ICR: institute of cancer research; NK: natural killer; 5-FU: 5-Fluorouracil; TNF-α: tumor necrosis factor-alpha; IL: interleukin; IL-1β: interleukin-1beta; iNOS: inducible nitric oxide synthase; COX-2: cyclooxygenase-2; NF-κB p65: nuclear factor-kappa B p65; p-MAPK: phosphorylated mitogen-activated protein kinase; BW: body weight; H_2_O_2_: hydrogen peroxide; MB: manganousethylenebis (dithiocarbamate); RBCs: red blood cells; WBCs: white blood cells; DNA: deoxyribonucleic acid; MDA: malondialdehyde; AOPP: advanced oxidation protein products; SOD: superoxide dismutase; GSH: glutathione; GPx: glutathione peroxidase; MCV: mean corpuscular volume; MCH: mean corpuscular hemoglobin; MCHC: mean corpuscular hemoglobin concentration; DNCB: 2,4-Dinitrochlorobenzene; IFNγ: interferone gamma; PTX: paclitaxel; PBMCs: peripheral blood mononuclear cells; κ: kappa; LMW: low molecular weight; HMW: high molecular weight; MPO: myeloperoxidase; LMWDPs: low-molecular-weight degradation products; CDK2: cyclin-dependent kinase 2; E2F2: E2F transcription factor 2; ↑: increased; ↓: decreased. * Inhibiting (IC_50_) the coagulation factor residual activity. ** Enabling the doubling of the coagulation time of the negative control (NaCl 0.9%).

### 5.2. Chondrus ocellatus

*Chondrus ocellatus* Holmes 1896 distributes naturally around coastal areas of South Korea, Japan, China, and Taiwan [110]. The first report of this species dates back to Japan [111]. *Chondrus ocellatus* Holmes 1896 can be found mainly in Korea and China and a few of its strains showed a presence in Japan. However, *C. ocellatus* f. *crispoides* Mikami 1965 is the main species from Japan. Previous studies have documented information pertinent to the life history, morphology, crossability, and growth of this red algal species [112,113]. In Korea, particularly, the habitat of this species comprises a lower intertidal zone of rocky shores [114]. During ancient times, the alga had a dual purpose as a viable food source and a medicinal remedy for ailments such as persistent constipation and bone fractures [115]. Akin to *C. crispus*, *C. ocellatus* is also a good source of carrageenan. Earlier, little information was available on the chemical constituents and nutrients of this species. But recently, the chemical composition (%) of ethanolic extracts of *C. ocellatus* was assessed using response surface methodology, considering three parameters: ethanol concentration, ultrasonic temperature, and ultrasonic time. The extract composition consisted of total phenolic content (24.8%), total sugar content (6.94%), protein (17.1%), ash (34.4%), and crude fiber (1.83%) [102]. 

More recently, the comprehensive lipid profile of *C. ocellatus* as natural biochemical markers using reversed-phase liquid chromatography coupled with quadrupole time-of-flight mass spectrometry was documented and described as qualitative and semi-quantitative lipid data [116]. Lipid composition (%) identified five major classes, including glycolipids (GLs, 56.3%), glycerolipids (GPs, 20.25%), betaine lipids (BL, 0.03%), phospholipids (PLs, 21.3%), and sphingolipids (SLs, 2.13%). These classes are further divided into 25 subclasses. The GLs’ subclasses include DGDG (7.27%), DGMG (0.05%), MGMG (0.52%), lyso-monogalactosyldiacylglycerol (MGDG, 18.18%), and SQDG (14.54%), with MGDG being the most abundant. Another subclass includes glucuronosyldiacylglycerols (GlcADG, 0.21%), acylated glucuronosyldiacylglycerols (acGlcADG, 0.47%), head-group acylated DGDGs (acDGDG, 1.04%), and head-group acylated MGDGs (acMGDG, 14.02%). GLs were found to be constituted of PUFAs, including AA and EPA, indicating the nutritional potential of this species. 

In the PL category, subclasses include phosphatidylcholine (PC, 9.87%), phosphatidylethanolamine (PE, 3.12%), phosphoglycerols (PG, 6.23%), and lysophospholipids, including lysophosphatidylglycerol (LPG, 0.05%), lysophosphatidylcholine (LPC, 1.56%), lysophophatidylethanolamine (LPE, 0.31%), and phosphatidylinositol (PI, 0.16%). Among BL, the presence of diacylglyceryl-N, N, N-trimethylhomoserine (DGTS, 0.03%) was observed. Cer (0.52%), HexCer (1.56%), and inositol phosphoryl ceramides (IPC, 0.05%) subclasses were observed in SLs. Notably, the SLs profile was marked by the presence of a tetrahydroxy long-chain base (LCB), dehydrophyto-sphingosine (t18:1), which was the most abundant LCB in the Cer and HexCer profile. In GPs, triacylglycerol (TG, 8.31%) and diacylglycerol (DG, 11.94%) have been identified from *C. ocellatus* [116]. The study highlights the importance of EPA and AA as important lipid biomarkers for the identification of red alga species, including *C. ocellatus*. A few biological applications of this species have been reported in the existing literature compiled in the subsequent sections.

#### 5.2.1. Anti-Tumor and Immunomodulation 

A single team of researchers has primarily evaluated the literature demonstrating the anti-tumor abilities of this species. As per one study [98], five λ carrageenans of different molecular weights (kDa) (PC1-650, PC2-240, PC3-140, PC4-15, and PC5-9.3), were prepared using the microwave approach. All of the extracts had anti-tumor and immune-modulatory effects on animals that had S180 and H22 tumor cells implanted. PC4 and PC5 showed the highest potential among all carrageenans [98] (Table 1). In a continuous effort, enhanced antitumor potential of a low-molecular-weight (9.3 kDa) fraction along with 5-fluorouracil (5-FU) was observed in a mouse model transplanted with the S180 tumor. Further, the co-treatment led to reduced side effects of 5-FU treatment [99]. Another study with similar experiments and findings was reported for low-molecular-weight carrageenan and 5-FU in a mouse model transplanted with H-22 tumor cells [100] (Table 1). These studies suggest the anti-tumor potential of *C. ocellatus*. The studies showcase the endeavors of a research group. No other investigations have been recorded thereafter. Therefore, further research should assess the potential of this species and its bioactive components as anticancer agents.

#### 5.2.2. Anti-Inflammatory Activity

Generally, inflammation is a natural, non-specific defense system of the body, but in certain circumstances, the process may have negative implications by attacking tissues within the body, leading to inflammatory diseases [117]. Its characteristic symptoms include heat, pain, redness, swelling, and function loss. Available anti-inflammatory drugs are not without side effects [118,119]. Therefore, efforts to regulate inflammation and its related complications are always focused on finding more natural solutions, including those derived from marine wealth [120].

Recently, the anti-inflammatory potential of the ethanolic extract of *C. ocellatus* was demonstrated in lipopolysaccharide-induced RAW cells (macrophage cell line) and croton oil-induced ear edema mice. In vivo inflammatory effects are most commonly evaluated using the percentage reduction in paw edema volume generated by carrageenan. The extract showed potential beneficial effects against inflammation by modulating nuclear factor-kappa B (NF-κB) and the mitogen-activated protein kinase (MAPK) signaling pathways [101] (Table 1).

#### 5.2.3. Anti-Diabetic and Antioxidant Activities 

Diabetes is a chronic metabolic condition marked by elevated glucose levels in the bloodstream due to malfunctions in the carbohydrate and lipid metabolisms. This chronic disease can lead to several macrovasulcar and microvascular complications [121]. Thus, research on plant-derived ingredients as potent anti-diabetic formulations is ongoing, despite the availability of anti-diabetic medications [122,123,124]. Moreover, research on marine seaweed components for managing diabetes has gained momentum in the last decade [125,126]. However, limited information is available on *Chondrus* spp. Zhu et al. [102] extracted potential polyphenolic compounds from *C. ocellatus* using response surface methodology and evaluated them for antioxidant and anti-diabetic properties in vitro. Following extract supplementation, enzymatic inhibition of α-amylase and α-glucosidase was identified as competitive and mixed inhibition modes, respectively, along with remarkable antioxidant activity [102] (Table 1). Till now, a single study has evaluated the anti-diabetic potential of *C. ocellatus* species based on in vitro enzymatic assays. More research is needed to evaluate different extract types in cell and animal models to harness the full potential of this species for managing diabetes and related complications. 

### 5.3. Mazzaella canaliculata (C. Agardh) Arakaki & M. E. Ramírez 2021 (Formerly Known as Chondrus canaliculatus)

*Mazzaella canaliculata* is an indigenous red marine alga species found throughout the temperate Pacific coast of South America [127]. The species can be located in the intertidal regions and are known producers of carrageenans. According to a recent study, this is the only species that is categorized as belonging to the Southern Hemisphere [32]. In contrast to *C. crispus*, limited information is available on the chemical constituents of this species. 

The mineral (mg/L) content of *M. canaliculata* includes major components, namely, Mg (1103.61 ± 0.11), Ca (676.55 ± 3.21), K (8027.11 ± 0.8), P (366.72 ± 6.12), Na (2474.11 ± 4.95), and minor components, including Fe (20.44 ± 2.92) and Zn (0.56 ± 0.56) [103]. The chemical composition (%) of polysaccharides from *M. canaliculata* based on dry weight, as reported by Jaballi et al. [104] consists of yield (2.05%), protein (1.57%), total sugars (49.42%), sulfate on the sugar backbone (0.0025), uronic acids (16.80), and ash (2.57%, based on wet weights) [104]. In the subsequent section, the health-promoting activities of *M. canaliculata* have been described. 

#### 5.3.1. Antioxidant and Antimicrobial Activities 

Xenobiotic compounds pose a threat especially by causing oxidative damage. Thus, researchers are looking for therapeutic options available, and the quest for compounds from marine algae has received significant attention. The therapeutic potential of *M. canaliculata* species against maneb (Manganousethylenebis (dithiocarbamate) MB, fungicide)-instigated oxidative stress has been evaluated recently [103]. The extract showed DPPH radical scavenging activity but was lower than vitamin E and BHT standards. In vivo, *M. canaliculata* extract improved the antioxidant defense mechanism and minerals in bone and erythrocytes in rats co-treated with MB and algal extract. The bone histo-architecture also showed improving signs. The study suggests the protective effects of a methanolic extract of *M. canaliculata* against MB-stimulated oxidative damage, hematotoxicity, and genotoxicity in blood and bone [103]. As per the authors, this study first reported the protective effects of *M. canaliculata*. The extract also reported antimicrobial activity against seven microbial strains.

#### 5.3.2. Nephro-Protective Activity 

Nephrotoxicity refers to the adverse impact of chemicals and medications on the normal physiological processes of the kidney [128]. The nephrotoxicity caused by drugs leads to severe medical conditions, including acute renal injury and chronic kidney disease [129]. Many studies have evaluated the nephroprotective effects of phytochemicals [130]. The same research group that documented cytoprotective effects, isolated polysaccharide from *M. canaliculata* and evaluated its antioxidant (in vitro) and nephroprotective activity in vivo. Different doses were evaluated against MB-induced nephrotoxicity in vivo. Co-treatment with polysaccharide led to improved biochemical parameters, hematological parameters, and the kidney’s oxidative injuries (Table 1). The study suggests the potential nephro- and hemato-protective action of *M. canaliculata* [104].

#### 5.3.3. Anti-Atopic Activity 

Atopic dermatitis (AD) is a common, inflammatory skin (chronic) disease that occurs due to many genetic, immunological, and environmental factors. It is characterized by the activation of various T cell types, disruption of the skin’s protective barrier, and an imbalance in the normal skin bacteria [131,132]. Seaweeds derived components have also been evaluated against AD [133,134]. In a 2,4-dinitrochlorobenzene-induced mouse model, Kang et al. examined the anti-atopic dermatitis properties of ethanolic extracts of the red alga *M. canaliculata* [105] in a mouse model induced by 2,4-dinitrochlorobenzene. As per the study, red algae extract treatment suppressed the atopic dermatitis symptoms, including degree of skin lesions. The extract supplementation reduced levels of pro-inflammatory cytokines, including interferon-γ and interleukin-4 [105] (Table 1). The study suggests the therapeutic potential of *M. canaliculata* extract against atopic dermatitis

### 5.4. Chondrus armatus 

*Chondrus armatus* (Harvey) Okamura 1930 was reported first from Dalian by Luan and Zhang [135]. *Chondrus armatus* has been exploited for the isolation of polysaccharides, namely κ- and κ/β-carrageenans [136]. The following section is going to describe the various biological applications of *C. armatus* extract and its carrageenans.

#### 5.4.1. Anti-Inflammatory Activity 

It is well known that Rhodophyta is a good source of carrageenans, including high-molecular-weight carrageenans. In a study, the high-molecular-weight (HMW, ~250 kg/mol) carrageenan was isolated from *C. armatus* and further acid-treated to obtain a low-molecular-weight (LMW, ~2.3 kg/mol) fraction. Both HMW and LMW fractions were evaluated against acetic acid-induced colitis in vivo. HMW fraction at a 5 mg/kg dose reduced the degree of colon damage more than twice and the area of damage by 40%, and it also suppressed (2.9 times) myeloperoxidase activity. While the remaining chosen doses of HMW fractions led to inflammation in animals (Table 1). No protective effect was observed at dose (10 mg/kg) for the HMW fraction, the acid-hydrolyzed fraction, or the naïve polysaccharide. The study corroborated the earlier finding that a high dose of polysaccharides can instigate inflammation. The research shows that the polysaccharide’s anti-inflammatory effects depend on the dose. It may work by reducing the number of neutrophils that enter the area and by acting as an antioxidant [106]. Thus, care must be taken about the dose amount and molecular weight of fractions from this red algal species used to treat colitis in the near future.

#### 5.4.2. Anti-Tumor and Immunomodulation Activity

In a recent study, κ- and λ-carrageenan polysaccharides and their low-molecular weight-products were assessed as anti-tumor and immunomodulators [107]. The study reported differences in the biological properties of HMW and their LMW degradation fractions. All tested polysaccharides decreased the metabolic activity of cancer cell lines, namely squamous cell carcinoma (KYSE30) and human esophageal adenocarcinoma (FLO1). The treatments did not lead to erythrocyte hemolysis or normal leucocytes or fibrocytes. In addition, all polysaccharides stimulated proinflammatory cytokine production, while anti-inflammatory cytokine (IL-10) was stimulated only after LMW λ-carrageenan treatment [107] (Table 1). Overall, the study indicates that low-molecular-weight degradation products have enhanced biological properties. The same research group looked at (HMW) κ- and λ-carrageenans from *C. armatus* and their low-molecular-weight breakdown products (LMWDPs) again. Among all, only LMWDPs of κ-carrageenan at a dose of 100 μg/kg/day for 7 days developed leukopenia in animals. The application of λ- and κ-carrageenan derived from *C. armatus* has been found to enhance the motility of macrophages while simultaneously reducing their phagocytic activity (Table 1). This characteristic renders these carrageenan compounds suitable for application as immuno-modulatory materials [108].

Another recent study demonstrated the tumor suppressive activity of λ- and κ-carrageenan polysaccharides from *C. armatus* against gastrointestinal cancer cell lines. Tumor suppression was found to be due to cytotoxicity and the inhibition of cell cycle progression. Treatment of κ-carrageenan delayed the S-phase stage in HCT-116 (colorectal carcinoma) and FLO-1 (esophagus cancer line) cells, while a noticeable delay at the G2/M stage was witnessed in KYSE-30 (esophagus cancer line) cells (Table 1). Of note, κ-carrageenan inhibited cell cycle progression in the G1 phase in RKO (colon carcinoma cell line) cells. Both carrageenans induced apoptosis in selected models, but a profound effect was observed in RKO cells by promoting early and late apoptosis. In the FLO-1 cell line, only κ-carrageenan showed an apoptosis-inducing ability [109]. Overall, a couple of studies pertaining to *C. armatus* shows the biological potential of this red algal species. More studies should be conducted in the future, considering cell and animal models, to realize the full potential of *C. armatus*.

## 6. Other Species and Biological Applications

In addition to summarizing the biological properties of four *Chondrus* species, a few studies on other *Chondrus* species have also been published in the literature. For instance, *C. pinnulatus* has duly recorded an inhibitory activity against cyclicAMP phosphodiesterase. The aqueous extract displayed an IC_50_ value of 3.3 μg/mL, inhibiting the enzyme [137]. Another study evaluated the anti-inflammatory potential of the ethanolic extract of *C. nipponicus* Yendo from the Korean Peninsula. Extract supplementation inhibited the expression of various inflammatory markers by modulating the NF-κB and MAPK signaling pathways [138]. The anti-inflammatory properties of *C. verrucosus* species have also been documented. The anion exchange chromatography was used to separate polysaccharides. This led to the identification of three fractions, which were named CV1 through CV3, based on how they eluted. Three components exhibited varying levels of sulfate concentration and demonstrated distinct anti-inflammatory effects in RBL-2H3 (basophilic leukemia cell line) cells [139]. 

## 7. Conclusions and Future Directions 

To summarize, *Chondrus* spp. possess a significant amount of nutrients and has been shown to have beneficial biological properties. It is documented to have various macronutrients, micronutrients, and bioactive compounds. Notably, higher amounts of PUFAs, including AA and EPA [63], MAAs, ascorbate, and α-tocopherol, have been reported, particularly in *C. crispus*. The studies have shown various health benefits for *Chondrus* spp. In addition to health benefits, *Chondrus* spp. is a good source of carrageenan and agar used for functional food ingredients, food additives, cosmetics, and pharmaceutical applications. Thus, it offers myriad benefits, either when directly consumed or supplemented in diets as a food additive. Most biological activities are presented as in vitro and in vivo studies, and a few health benefits are evidenced based on a single study. Further, the data from the clinical study and bioavailability of *Chondrus* spp. components is inadequate. Despite its importance, the genus is underpinned and demands extensive evaluation for more health-promoting and pharmacological effects. 

The efficacy of *Chondrus* extracts as neuroprotective and stress-alleviating agents has been proven in a worm model. Future studies should focus on evaluating their effects on humanized cell lines (such as HMC3 cells) and animal models for Alzheimer’s and Parkinson’s diseases, should target ROS mitigating signaling pathways by utilizing various bioactive constituents from *Chondrus*. The gut microbiota in individuals undergoes evolutionary changes over their lifetime and has a crucial role in both health and disease. The presence of gut dysbiosis has been identified as an important factor that interacts with human metabolism in diverse ways, hence contributing to the development of numerous pathological conditions. Research is ongoing into many natural components to restore the gut microbiota by targeting chronic diseases. The beneficial effects of seaweed components on human gut microbiota have been documented. Seaweed polysaccharides have been found to effectively modify the composition of gut bacteria by functioning as prebiotics. This leads to the modulation of gut bacteria and the production of SCFAs [140]. However, there is only one study available on cultivated *C. crispus*. Thus, future studies should first focus on cell and animal studies, and then later on large-scale clinical studies evaluating the effects of its bioactive constituents including polysaccharides (carrageenan) as gut microbiota modulators. Researchers have found that carrageenan from red seaweeds can help improve the symptoms of metabolic syndrome by changing the gut microbiota [141] and also by acting as potential antitumor agents [142]. However, safety concerns of carrageen should be thoroughly investigated [143]. Future research should focus on evaluating the effects of *Chondrus* species and its bioactive constituents on different pathological conditions such as obesity, diabetes, inflammatory disease, and cancer targeting the gut microbiota. 

Although the chemical composition of different species has been documented from different extracts, moreover, in-depth quantity–effect relationships still need to be further assessed. The presence of novel compounds in seaweeds is largely influenced by their evolutionary origin, harvest from wild or land-based IMTA systems, climatic conditions, conservation, and extraction procedure including the solvent type. Thus, more sophisticated methods should be employed to extract and identify novel chemical compounds with improved biological activity. As stated before, a few studies have documented the use of new extraction technologies. In the near future, it is suggested to consider employing non-destructive approaches, including surface-enhanced Raman spectroscopy, hyperspectral imaging, near-infrared spectroscopy, terahertz spectroscopy (consisted of electromagnetic waves of 30 μm to 3 mm wavelength range), and fluorescence spectroscopy for evaluating compounds from *Chondrus* spp. [144,145]. Furthermore, extractions should be focused on the chemical composition of both wild and cultivated *Chondrus* species. On-land cultivation should be promoted for consistent supply and food security and to avoid conflicts on the marine territory. The development and use of highly efficient extraction techniques are needed to quantify different algochemical profiles under different annual ecological conditions. More mechanistic studies are needed to determine the potential targets for genus *Chondrus* and obtain a better picture of its therapeutic potential. 

As mentioned in the review, Song et al. [116] assessed the lipidomic profile of *C. ocellatus*. The study evaluated and compared lipid profiles of four different red seaweed species and documented a few novel lipid compounds that added knowledge on sphingolipids and glycolipids of seaweeds, and added knowledge about the unique and natural fingerprinting profile. Subsequent investigation should rely on lipid quality metrics and assess new molecules for diverse biomedical applications. Further, more such studies are needed for other *Chondrus* species, both from wild and cultivated regimes, as well as the application of transcriptomics, metabolomics, and proteomics, which will be necessary in the future to gain further insights into this seaweed.

Functional genomics is an important approach for gaining a comprehensive understanding of pathways of metabolism and identifying and characterizing genes responsible for the production of important industrial products and by-products should be applied to have a better understanding of *Chondrus* spp. Collén et al. reported important information on the genome structure (105 Mbp size) of *C. crispus* and offered valuable insights into the origin and evolution of this red algal species. The study sheds light on the metabolism and marine adaptations of this species. Notably, the study highlights the gene sets associated with carbohydrate metabolism, including those connected to starch biosynthesis and cellulose synthases [146]. 

International collaborations are necessary among primary and secondary producer countries of *Chondrus* spp. to promote and develop agricultural technology and disseminate knowledge. In addition to playing role in human health promotion, this genus has the potential to be utilized as a biostimulant in the horticultural and agricultural sectors. Recently, the protein-rich extract of *C. crispus* was first used as biostimulant to enhance the growth and drought tolerance of tomato plants [147].

Carrageenan production from *Chondrus* spp. is a well-known commercial application of this genus. The potential of this genus should be explored for other bio-based products and biofuels [148,149]. In one study, hydrothermal liquefaction (10 min, 345 °C, 16.7% solids) of biomass with a solvent (1:5) ratio yielded an 8% bio-yield from *C. crispus* [149]. Future research should explore the potential of biofuel generation using *Chondrus* spp. Further, carrageenan from *Chondrus* should also be evaluated in drug delivery, heavy metal quenchers, microcapsules, or microsphere applications. 

Given the nutritional significance of the *Chondrus* genus, may this red alga be classified as a nutritious superfood? Undoubtedly, the demand for seaweed as a superfood is increasing. Several obstacles must be addressed before *Chondrus* can be considered a viable food source in the future. The primary concern revolves around the safety of food, encompassing both microbiological and chemical hazards. Microbial safety focuses on the presence of pathogenic microorganisms in the areas where harvesting and cultivation take place. *Chondrus* spp. may harbor multiple potentially hazardous components that require careful attention. Another concern is the occurrence of allergic reactions. In general, seaweeds are not typically regarded as allergenic species. Nevertheless, a study regarding the allergic response to red algae has been documented [150]. It is important to assess the potential allergic reactions associated with *Chondrus* spp., which is a type of red alga. 

Another important aspect is acknowledging the organoleptic characteristics and overall consumer perception due to the unfamiliarity of this genus. Typically, the taste of seaweed is well received when combined with conventional cuisine. Therefore, it should be blended with other food groups in order to capture consumers’ interest. Furthermore, the utilization of processing technology to enhance nutritional content, optimize quality, preserve food, and conduct sensory analysis are additional challenges.

Overall, the genus is blessed to have many essential nutrients and health-promoting activities. Future research should prioritize several factors, such as advancing processing technologies for both wild and farmed species, exploring innovative extraction methods, and generating safe food products. Additionally, it should involve conducting cell and animal studies that may be translated into clinical studies to better understand the underlying molecular pathways. This could potentially contribute to the prevention of human diseases and the creation of functional food products. 

## Data Availability

Not applicable.
